# DSGF-Net: A Lightweight Dual-Stream Gated Fusion Network for Cross-Subject fNIRS Motor Task Classification

**DOI:** 10.3390/s26175401

**Published:** 2026-08-26

**Authors:** Jingfu Wu, Xiu Zhang, Xin Zhang, Deping Huang

**Affiliations:** 1 Tianjin Key Laboratory of Wireless Mobile Communications and Power Transmission, Tianjin Normal University, Tianjin 300387, China; haoshuaishuaiwo@163.com (J.W.); ecemark@tjnu.edu.cn (X.Z.); 18653874610@163.com (D.H.); 2 College of Artificial Intelligence, Tianjin Normal University, Tianjin 300387, China

**Keywords:** brain–computer interface, functional near-infrared spectroscopy, motor task classification, dual-stream network, gated fusion

## Abstract

Functional near-infrared spectroscopy (fNIRS) has become an important signal source in motor imagery (MI) brain–computer interface research due to its non-invasive nature and high application flexibility. However, fNIRS signals exhibit significant inter-subject variability, complementary information from oxygenated hemoglobin (HbO) and deoxygenated hemoglobin (HbR), and complex spatiotemporal dynamics, making their efficient and robust classification challenging. To address these issues, this paper proposes a Dual-Stream Gated Fusion Network (DSGF-Net). This model employs a dual-branch architecture to perform complementary feature modeling of fNIRS signals: one branch focuses on extracting multi-scale temporal dynamic features, while the other learns the spatial distribution of hemodynamic features across channels, thereby effectively characterizing the signals from different perspectives. Upon this foundation, a gated fusion mechanism was designed to adaptively adjust the importance of different feature dimensions after the fusion of the two feature streams, thereby enhancing the discriminative power of the fused representation. On two public datasets, MI and UFFT, experimental results based on leave-one-subject-out (LOSO) cross-validation show that the proposed method achieves competitive performance across metrics such as classification accuracy, F1-score, and Kappa coefficient. Furthermore, a comparative analysis of performance under different network component configurations validates the contributions of the dual-branch structure and the gated fusion mechanism to performance improvements. Furthermore, complexity analysis results show that DSGF-Net achieves superior classification performance while maintaining a relatively small parameter size, striking a good balance between performance and computational complexity. DSGF-Net provides an effective, lightweight deep learning framework for offline fNIRS-based motor task classification, with potential applications in cross-subject BCI systems and brain signal decoding.

## 1. Introduction

Brain–computer interfaces (BCIs) are a groundbreaking human–computer interaction technology designed to establish a direct communication channel between the brain and external devices, offering significant practical value in fields such as neurorehabilitation and assistive control. Among the various BCI paradigms, motor imagery (MI) has become a hot research topic because it does not require external stimuli and aligns with humans’ natural motor cognitive processes. When a user performs or imagines a limb movement, specific neural activity patterns emerge in the motor-related cortical regions of the brain. By decoding these neural activities, BCI systems enable mind-controlled operation of external devices [1]. Functional near-infrared spectroscopy (fNIRS), as a non-invasive neuroimaging technique, has garnered widespread attention in the BCI field in recent years. This technology is based on the neurovascular coupling mechanism [2]; by measuring changes in the propagation of near-infrared light through the scalp and skull, it indirectly reflects the dynamic changes in oxygenated hemoglobin (HbO) and deoxygenated hemoglobin (HbR) in the cerebral cortex, thereby enabling the characterization of brain functional activity [3,4,5]. Although the hemodynamic response reflected by fNIRS exhibits a certain delay compared to electrophysiological signals, it provides a unique perspective for studying brain functional activity [6]. Compared to the more established electroencephalography (EEG), fNIRS provides complementary information related to hemodynamics [7,8]; at the same time, compared to functional magnetic resonance imaging (fMRI), it offers greater portability and lower cost, making it more suitable for BCI applications in natural settings [9]. However, due to the inherent delay in hemodynamic responses and the susceptibility of the signal to various physiological and motion artifacts, the effective decoding of fNIRS signals remains challenging [10].

In fNIRS-based motor imagery BCI research, signal classification methods remain a core factor influencing system performance. How to effectively model the spatiotemporal characteristics of signals and improve classification performance is one of the key issues in current research [9]. fNIRS signals typically contain both temporal dynamics and spatial distribution information and exhibit significant inter-individual variability. Furthermore, while both HbO and HbR can reflect cerebral hemodynamic responses from different perspectives and provide complementary information, it remains challenging to develop effective models that incorporate the spatiotemporal characteristics of fNIRS signals while fully preserving the information from them [11]. Existing studies have explored this topic from various perspectives, including temporal, spatial, and spatiotemporal feature extraction. However, there is still room for improvement in jointly modeling dynamic patterns across different temporal scales, as well as spatial and hemodynamic information, while fully leveraging the complementary nature of structural biases in different feature extraction methods. Therefore, how to establish an effective feature extraction and fusion mechanism to enhance the model’s ability to characterize complex fNIRS signals remains an important research question.

To address the above issues, this paper proposes a Dual-Stream Gated Fusion Network (DSGF-Net) for classifying fNIRS signals in motor tasks. This method employs a dual-stream architecture with different structural biases to model complementary features. Specifically, the Lightweight Multi-Scale Temporal-Dominant Stream (LMT Stream) focuses on extracting multi-scale, time-dominant spatiotemporal features and incorporates spatial information through lightweight cross-channel operations; the Spatio-Hemodynamic Stream (SH Stream) focuses on learning spatial structures and hemodynamic-related features from channel-level information. Through this architectural design, the two branches characterize fNIRS signals from different yet complementary perspectives, providing distinct feature representations for subsequent feature fusion. Subsequently, a gated fusion mechanism is used to weight the fused feature dimensions, thereby integrating the complementary information extracted by the two branches. Building on this foundation, a gated fusion mechanism is introduced to adaptively weight and fuse the features extracted from different streams based on the input sample, thereby improving the discriminative power and robustness of the feature representation.

The main contributions of this paper are as follows:

1. We propose a dual-stream feature learning framework, DSGF-Net, for learning complementary representations from fNIRS signals. The proposed framework employs dual-stream feature extraction modules with different structural biases to learn fNIRS representations from two complementary perspectives, namely temporal dynamics and spatial hemodynamic distributions, thereby enhancing the representational capacity of fNIRS features.
2. An adaptive feature fusion module based on a gating mechanism was designed to perform a dynamically weighted fusion of features extracted from different branches. This module can dynamically learn and assign importance weights to features from different brain regions and temporal locations, thereby highlighting task-relevant key spatiotemporal features while suppressing redundant information and noise interference, thus enhancing the model’s robustness to individual variations and noise interference.
3. Extensive experiments were conducted on two publicly available motion-related datasets. The results demonstrate that the proposed method achieves an optimal balance between model complexity and classification performance, outperforming various classical deep learning and lightweight baseline methods.

The remainder of this paper is organized as follows: Section 2 reviews the relevant literature; Section 3 introduces the proposed DSGF-Net method; Section 4 describes the datasets, evaluation methods, and experimental setup used in the experiments; Section 5 presents and analyzes the experimental results; and Section 6 summarizes the paper.

## 2. State of the Art

In brain–computer interface (BCI) research based on functional near-infrared spectroscopy (fNIRS), signal classification methods have evolved from traditional machine learning approaches to deep learning methods. Early research primarily relied on a combination of manual feature engineering and shallow classifiers. During the feature extraction stage, researchers typically derived time-domain and frequency-domain features from fNIRS signals, such as statistical features (e.g., mean, variance, peak, and slope) and time–frequency features (e.g., power spectral density and wavelet transform coefficients) [12,13]. These features were then fed into classifiers such as Support Vector Machines (SVMs), Linear Discriminant Analysis (LDA), or k-Nearest Neighbors (k-NN) for classification. While such methods offer the advantages of simple structure and high interpretability, their feature representation and generalization capabilities remain somewhat limited when dealing with high-dimensional, nonlinear fNIRS signals that exhibit significant inter-individual variability. With the advancement of deep learning, researchers have begun to utilize neural network architectures for end-to-end modeling of fNIRS signals to automatically learn latent spatiotemporal feature representations and perform classification. Convolutional Neural Networks (CNNs), due to their advantages in local feature extraction, have been widely applied to fNIRS signal classification tasks [14,15]. For example, Sarhan et al. employed a CNN architecture for feature learning and classification of fNIRS signals, extracting local spatiotemporal patterns through convolutional operations, and achieved good recognition performance in a motor imagery task [16]. Meanwhile, Recurrent Neural Networks (RNNs) and their variant, Long Short-Term Memory (LSTM) networks, have been employed to model the temporal dependencies in fNIRS signals. Relevant studies indicate that LSTMs can, to a certain extent, capture long-term dependency information in sequential data through their gating mechanisms, thereby improving classification performance [17]. In recent years, Transformer architectures based on attention mechanisms have also been gradually introduced into the field of fNIRS signal analysis to model global dependencies and enhance feature representation capabilities [18]. For example, the fNIRS-T model proposed by Wang et al. achieved modeling of global contextual information by introducing Transformer structures at both the spatial and channel levels, yielding favorable experimental results on public datasets [19]. However, Transformer and deep CNN models typically rely on high model complexity and are prone to overfitting in scenarios with small fNIRS datasets. Therefore, some studies have attempted to improve model training stability and generalization ability by introducing regularization strategies (such as Dropout and Flooding) [20,21]. In addition to optimizing network architecture, some studies have begun exploring modeling methods that incorporate the physiological characteristics of fNIRS signals to enhance the model’s ability to capture hemodynamic patterns. Several lightweight models have achieved competitive performance while maintaining low model complexity by incorporating prior information related to hemodynamic responses [22]. For example, the lightweight fNIRS model proposed by Zenghui Wang et al. incorporated knowledge of delayed hemodynamic responses into its network design and achieved favorable experimental results on public datasets [23]. Furthermore, multi-scale convolutional structures have been employed for fNIRS feature extraction; by using convolutional kernels with different receptive fields to capture signal changes across various temporal scales, these structures enhance the expressive power of the features [24].

To fully leverage the complementary information among different types of features, multi-branch networks and dual-stream networks have gradually been applied to complex pattern recognition tasks. Compared to single-path networks, multi-branch architectures can learn latent representations in the data from multiple perspectives by designing network branches with different receptive fields and feature extraction preferences. For example, the Inception architecture in GoogLeNet extracts features at different scales through parallel convolutional branches, effectively enhancing the network’s ability to model multi-scale patterns [25]. Dual-stream networks were applied to video understanding tasks; by modeling spatial information and temporal dynamics separately and fusing features in the back end, they achieve the joint utilization of complementary information [26]. Similar ideas have gradually been introduced into the field of brain signal analysis. Given that electroencephalogram (EEG) signals exhibit both temporal dynamics and spatial topological structures, some studies have adopted dual-branch or multi-branch convolutional architectures to learn information representations in different dimensions separately and obtain more robust feature representations through fusion modules [27]. In fNIRS analysis tasks, since the signals simultaneously contain hemodynamic changes and spatial response patterns across different brain regions, multi-branch architectures provide an effective approach for fusing different feature representations. However, existing methods typically focus on enhancing feature extraction capabilities, while in-depth research on how to dynamically adjust the contributions of features across different branches based on input samples remains lacking.

How to effectively fuse features from different sources is also a key factor affecting the performance of deep models. Traditional fusion methods typically employ feature concatenation or fixed-weight fusion strategies; while these methods are simple to implement, they struggle to dynamically adjust the importance of different feature representations in response to changes in input samples. Gating mechanisms offer an effective solution to the aforementioned issues. Initially, gating structures were widely applied in recurrent neural networks; for example, Long Short-Term Memory (LSTM) networks control information flow through input, forget, and output gates, thereby enhancing the model’s ability to capture sequential information [28]. In recent years, the concept of gating has been further extended to multimodal learning and feature fusion tasks, enabling selective information processing and dynamic fusion by learning the weight distributions of different feature sources [29]. In the field of multi-source neural signal analysis, gating-based fusion methods can adaptively adjust the importance of different modalities or network branches based on the feature distributions of different input samples, thereby reducing redundant information and improving model robustness. However, in fNIRS-based motor imagery classification tasks, existing research has paid little attention to how to utilize gating mechanisms to coordinate the information contributions from different feature learning pathways. This is particularly true in cross-subject scenarios, where significant hemodynamic differences exist among participants, thus necessitating more flexible feature fusion strategies.

Overall, existing research has made some progress in fNIRS signal classification tasks, but several issues warrant further investigation. First, fNIRS signals typically contain both oxygenated hemoglobin (HbO) and deoxygenated hemoglobin (HbR) components. While these signals reflect different sources of hemodynamic information to some extent, there is still no unified and effective modeling approach for effectively leveraging their complementary nature. Among existing methods, some studies analyze fNIRS signals using a single modality or fuse them through simple concatenation, which makes it difficult to fully exploit the synergistic relationships among multi-source information. Second, fNIRS signals exhibit distinct spatiotemporal characteristics, containing both dynamic information that varies over time and specific spatial distribution patterns. Although previous studies have explored methods for modeling spatiotemporal features from various perspectives, achieving more effective feature representation across different scales and under different structural biases remains an ongoing research challenge. Particularly in multi-branch network architectures, the contribution of features extracted from different branches to the final classification task may vary dynamically depending on the input samples. Therefore, designing an adaptive feature fusion mechanism to enhance the model’s representational capacity and robustness for complex signals remains of significant research importance. Based on the above analysis, this paper proposes a Dual-Stream Gated Fusion Network (DSGF-Net). This method utilizes a dual-stream architecture to extract complementary feature representations from spatiotemporal convolutions with different structural biases and employs a gating mechanism to adaptively weight and fuse features from different branches, thereby enhancing the model’s ability to model key spatiotemporal patterns in fNIRS signals and improve its classification performance.

## 3. Methods

### 3.1. Overview of the Dual Stream Gated FusionNet Model

**Figure 1:**
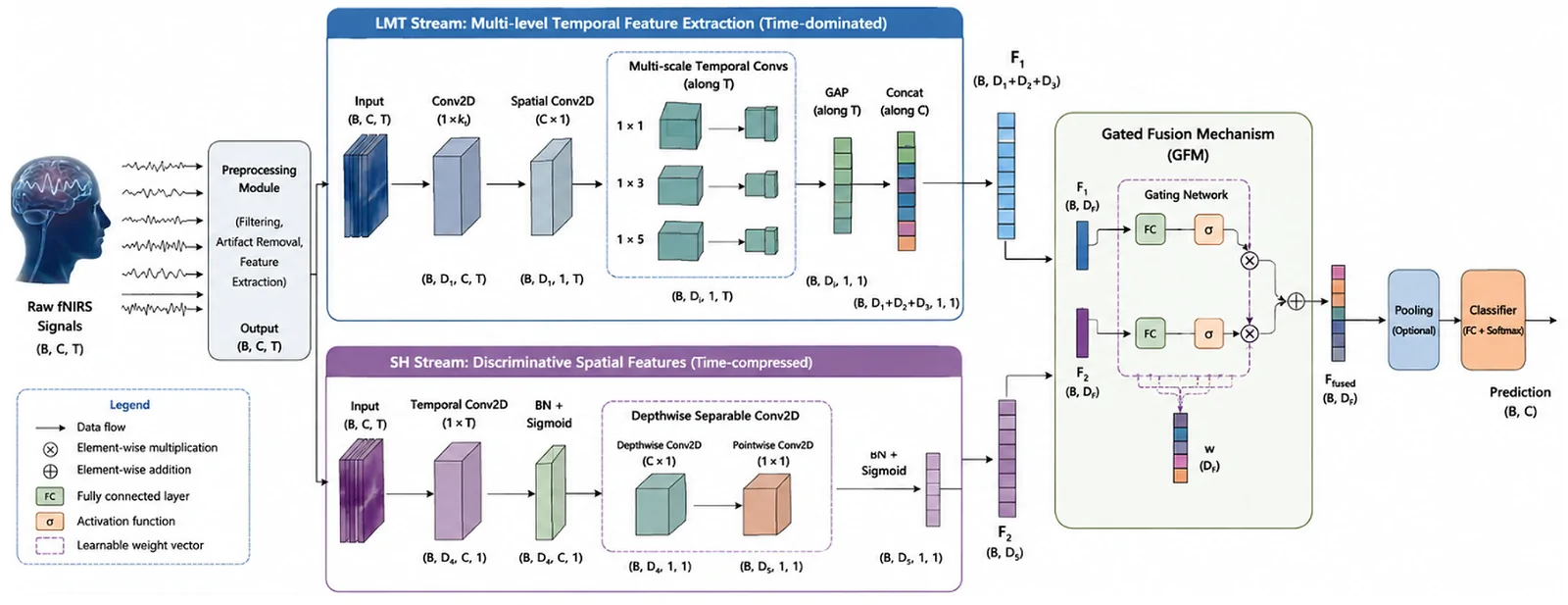
Schematic diagram of the DSGF-Net model.

This paper proposes a Dual Stream Gated FusionNet (DSGF-Net) for the classification of fNIRS motion signals. As shown in Figure 1, the model consists of a preprocessing module, a dual-branch feature extraction module, and a gated fusion classification module. Through feature learning with structurally biased approaches and an adaptive fusion mechanism, it effectively models the spatiotemporal features of fNIRS signals. At the input stage, the raw fNIRS signals first undergo a standard preprocessing workflow, including signal filtering, artifact removal, and basic feature construction, to reduce noise interference and improve signal quality. The preprocessed data are fed into the dual-stream feature extraction network to learn the spatiotemporal representations of the fNIRS signals from different perspectives.

Among these, Lightweight Multi-Scale Temporal Stream (LMT Stream) is primarily used to capture hemodynamic dynamics through multi-scale temporal convolution and, by combining channel relationship modeling, form a spatiotemporal feature representation. This branch models the dynamic changes across different temporal scales in the fNIRS signal through temporal convolution operations with varying receptive fields, thereby enhancing the model’s ability to represent hemodynamic response patterns. Meanwhile, the Spatio-Hemodynamic Stream (SH Stream) is primarily used to learn spatial distribution features across channels; by modeling the relationships between channels in different brain regions, it captures spatial activation patterns associated with motor imagery tasks. However, these two branches are not entirely independent, single-dimensional feature extraction modules; rather, they complement each other in modeling fNIRS spatiotemporal information based on different structural design biases, thereby enhancing the diversity and robustness of feature representation. The LMT Stream focuses more on modeling dynamic changes across different time scales, while the SH Stream emphasizes the ability to represent cross-channel spatial structural information. Subsequently, the feature representations ($F_{1}$ and $F_{2}$) output by the two branches are concatenated along the feature dimension and fed into the gated fusion mechanism (GFM) module. This module learns adaptive weight assignments along the feature dimension to perform weighted fusion of features from different sources, generating Fcomb, thereby enhancing the ability to select discriminative information and suppressing redundant features. This mechanism can dynamically adjust the fusion strategy based on the feature distributions of different input samples, thereby improving the model’s stability and generalization ability across different subjects. Finally, the fused feature representation undergoes dimensionality reduction via global average pooling and is fed into a fully connected classifier to predict the category of the motor imagery task.

### 3.2. Lightweight Multi-Scale Temporal-Dominant Stream

**Figure 2:**
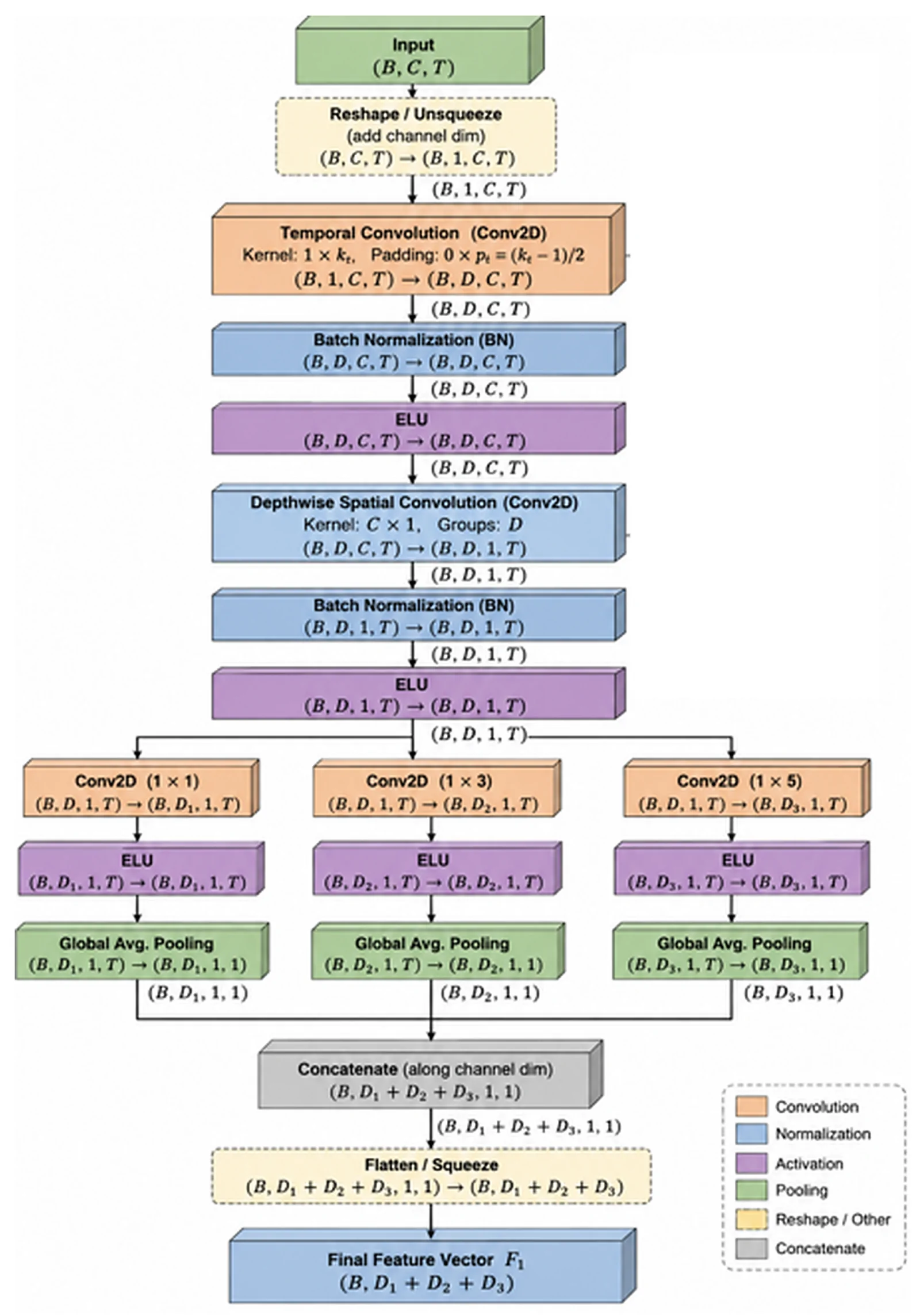
Schematic diagram of the LMT Stream model.

LMT Stream is a key submodule within DSGF-Net, designed to extract spatiotemporal feature representations dominated by temporal dynamics with low computational complexity. Given that fNIRS signals exhibit both pronounced temporal dynamics and spatial channel correlations, LMT Stream is not, strictly speaking, a pure temporal feature extraction module; rather, it prioritizes modeling temporal variation patterns while further incorporating information interactions between channels in different brain regions through lightweight spatial convolution. As shown in Figure 2, the input to the LMT Stream is represented as a three-dimensional tensor with shape (B,C,T), where B represents the batch size, C represents the number of fNIRS channels, and T represents the length of the time series (time steps). To accommodate 2D convolution operations, a convolution channel dimension is first added to the input tensor, restructuring it from (B,C,T) to (B,1,C,T), thereby fully preserving the original channel–time 2D structure. This operation merely changes the tensor’s representation; it does not mix or disrupt the structural information between the channel and time dimensions. During the temporal feature modeling stage, LMT Stream first uses a 2D convolution kernel of size $1\times k_{t}$ to extract temporal features from the input, where $k_{t}$ represents the size of the temporal convolution kernel. Since the kernel has a size of 1 in the channel dimension, this operation primarily extracts local features along the time axis T without spatially mixing features across different fNIRS channels. By introducing zero-padding along the temporal dimension $p_{t}=\left(k_{t}-1\right)/2$, the temporal dimensions before and after convolution are kept consistent. Specifically, the input tensor (B,1,C,T) to (B,D,C,T), where D represents the number of feature channels after temporal convolution. Subsequently, normalization is performed via a Batch Normalization (BN) layer, and nonlinearity is introduced using the Exponential Linear Unit (ELU) activation function. After completing time-domain local feature extraction, LMT Stream further employs depthwise spatial convolution to model the spatial correlations among different fNIRS channels. Specifically, a depthwise convolution kernel of size C×1 is used, and the data are grouped by feature channel so that each feature channel independently aggregates information across fNIRS channels. This operation transforms the feature tensor changes from (B,D,C,T) to (B,D,1,T), thereby preserving the temporal dimension while fusing spatial channel information. Compared to standard convolution, this spatial convolution uses a depthwise approach to group channels, which can significantly reduce the number of parameters and computational load. After spatial convolution is complete, the features undergo BN and ELU again for normalization and nonlinear transformation. To further enhance the model’s ability to capture patterns of change across different time scales, LMT Stream subsequently employs three parallel multi-scale convolution branches, using 1×1, 1×3, and 1×5 convolution kernels, respectively. Since spatial convolution has already reduced the spatial dimension to 1, these three convolution branches primarily use different receptive fields to extract features along the remaining temporal dimension. For the i-th branch, its output can be expressed as $F_{i}\in R^{B\times D_{i}\times 1\times T}$. Here, $D_{i}$ represents the number of feature channels in the output of the i-th branch. The three branches are used to capture local variation patterns at different time scales; smaller convolution kernels focus on rapid local changes, while larger convolution kernels cover a wider temporal receptive field, thereby enhancing the model’s ability to represent hemodynamic variation patterns across different time scales. Subsequently, each branch undergoes feature compression via global average pooling (GAP). Since the previous spatial convolution stage has already reduced the spatial dimension to 1, GAP is applied to the remaining 1×T feature map and compresses the features of the i-th branch from $\left(B,D_{i},1,T\right)$ to $\left(B,D_{i},1,1\right)$. The outputs of the three branches are then concatenated along the feature channel dimension, yielding $\left(B,D_{1}+D_{2}+D_{3},1,1\right)$, and the final feature vector is obtained through a flattening operation $F_{1}\in R^{B\times (D_{1}+D_{2}+D_{3})}$. Rather than modeling features in a single dimension in isolation, LMT Stream incorporates channel-dimension information into a time-dominated modeling process through different convolution scales and structural biases, thereby achieving a comprehensive representation of the spatiotemporal features of fNIRS signals. The final output features—combining $F_{1}$ with features extracted from another branch—are fed into a gated fusion module to enable adaptive feature fusion and classification decisions.

### 3.3. Spatio-Hemodynamic Stream

**Figure 3:**
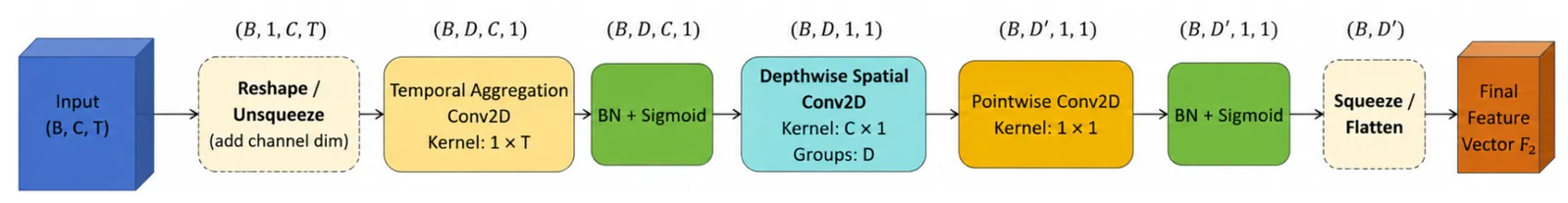
Schematic diagram of the SH Stream model.

The SH Stream is another core branch module in DSGF-Net, primarily used to learn spatial structural information along the channel dimension from fNIRS signals and to extract discriminative features related to the motor imagery task. As shown in Figure 3, this module also takes a 3D tensor of shape (B,C,T) as input, where B represents the batch size, C represents the number of fNIRS channels, and T represents the time step. In the initial stage of feature modeling, to compress and represent the overall trend of changes across the temporal dimension, the input signal is first processed through a temporal convolution operation. This convolution uses a kernel size of 1×T to globally aggregate the time-series information within a single channel, thereby obtaining a preliminary representation of the overall temporal dynamics. Subsequently, the feature distribution is normalized using Batch Normalization (BN) to improve training stability, and a sigmoid activation function is applied to introduce a nonlinear transformation, thereby enhancing the expressive power of the features. After obtaining the temporally aggregated representation, SH Stream proceeds to the spatial feature modeling stage to capture the structural relationships among different fNIRS channels. This stage employs a depthwise separable convolution architecture for efficient modeling. Specifically, depthwise convolutions are first used to process the features of each channel independently. The convolution kernel size is set to C×1 to reorganize features and aggregate information along the channel dimension, thereby modeling cross-channel correlation patterns. Subsequently, the channel-level features are linearly combined and fused through pixel-wise convolution with a kernel size of 1×1 to generate a higher-level spatial feature representation. This structure helps enhance the model’s ability to capture feature interactions across different channels while maintaining low computational complexity. After spatial feature fusion, the features undergo normalization and nonlinear mapping via a Batch Normalization (BN) layer and a sigmoid activation function to stabilize the feature distribution and enhance expressive power. Finally, this branch outputs a feature vector $F_{2}$, which represents cross-channel structural information and task-related patterns in the fNIRS signal. The SH Stream does not independently model “pure spatial features” in the strict sense, but rather enhances the expression of spatial structural information through feature reorganization along the channel dimension, based on a time-compressed representation. This design complements the LMT Stream, and together they provide multi-perspective feature representations for the subsequent gated fusion module. Ultimately, the feature $F_{2}$ output by the SH Stream is fed into the gated fusion module alongside the multi-scale temporal feature $F_{1}$ extracted by the LMT Stream to achieve adaptive feature fusion and optimized classification decisions.

### 3.4. Gated Fusion Mechanism Module

The gated fusion mechanism (GFM) is a lightweight feature recalibration module in DSGF-Net. Its objective is to adaptively adjust the importance of different feature dimensions after the fusion of dual-stream features, thereby enhancing the discriminative power of the fused representation. Unlike explicit branch-level weight allocation strategies, the GFM proposed in this paper does not directly learn the weight relationships between the LMT Stream and SH Stream branches. Instead, it learns sample-relevant importance weights in the fused feature space based on the current input features, thereby enhancing discriminative feature responses and suppressing redundant information. After obtaining the output features $F_{1}$ and $F_{2}$ from the two branches, they are first concatenated along the feature dimension to obtain the combined feature representation $F_{\mathrm{comb}}$:

(1) $$F_{\mathrm{comb}}=\mathrm{Concat}\left(F_{1},F_{2}\right)$$

To achieve dynamic adjustment of the feature weights, we employed a nonlinear gating unit. We used a lightweight gating unit to adaptively recalibrate the joint features. This unit maps the joint features through a fully connected layer and, in combination with the sigmoid activation function σ, generates a weight vector *G* in the feature dimension. Its mathematical expression is as follows:

(2) $$G=\sigma (W_{g}\cdot F_{\mathrm{comb}}+b_{g})$$

In this model, $W_{g}$ and $b_{g}$ are learnable parameters, and the sigmoid function maps the output value range to the interval (0, 1), thereby assigning a continuous weight to each feature dimension that lies between full preservation and full suppression. After obtaining the gating weights, the combined features are reweighted through element-wise multiplication to obtain the final fused feature $F_{fused}$, expressed as

(3) $$F_{\mathrm{fused}}=F_{\mathrm{comb}}⊙G$$

This mechanism is essentially an adaptive recalibration strategy based on feature dimensions, designed to enhance the response to discriminative features while reducing the influence of redundant or weakly correlated features. This fusion method does not introduce additional complex attention structures; rather, it is a lightweight feature selection mechanism with low computational overhead. Finally, the fused features Ffused are fed into a classifier consisting of a Dropout layer and a fully connected layer to perform category prediction for motion tasks.

### 3.5. Label Smoothing

To improve training stability and alleviate over-confident predictions during model optimization, this study adopts label smoothing as an auxiliary regularization strategy, this study introduced the label smoothing regularization technique [30]. The core idea of this strategy lies in breaking the absoluteness of “hard targets” in traditional classification tasks, preventing the model from making overconfident predictions based on training labels and thereby mitigating the risk of the model overfitting to specific category-specific noise or biases. After the gated fusion module, the fused feature representation $F_{\mathrm{fused}}$ is fed into the final fully connected classifier to generate the raw classification outputs. Specifically, the classifier performs a linear transformation:

(4) $$Z=W_{c}F_{\mathrm{fused}}+b_{c}$$

where $W_{c}$ and $b_{c}$ denote the learnable weights and bias of the classification layer, respectively. $Z=[z_{1},z_{2},\ldots ,z_{C}]$ represents the output logits before probability normalization, where C is the number of target classes and $z_{i}$ indicates the unnormalized response corresponding to the i-th class. Unlike probability values, logits can take arbitrary real values and directly reflect the relative preference of the network toward different classes. To convert logits into a probability distribution, the Softmax function is applied:

(5) $$p_{i}=\frac{e^{z_{i}}}{\sum_{j=1}^{C}e^{z_{j}}}$$

where $p_{i}$ denotes the predicted probability of class i. The Softmax operation normalizes the logits into a probability vector $P=[p_{1},p_{2},\ldots ,p_{C}]$. Let $Y=[y_{1},y_{2},\ldots ,y_{C}]$ represent the original one-hot label vector. For the ground-truth class, $y_{i}=1$, while $y_{i}=0$ for all other classes. Instead of using the original hard target distribution, label smoothing constructs a soft target distribution $q_{i}^{\prime }$:

(6) $$q_{i}^{\prime }=\left(1-\varepsilon \right)y_{i}+\frac{\varepsilon }{C}$$

where ε represents the smoothing coefficient. The correct class is assigned a confidence weight of 1−ε, while the remaining uncertainty is uniformly distributed among all classes. The classification loss based on the original one-hot label is defined as

(7) $$L_{nll}=-\sum_{i=1}^{C}y_{i}\log\left(p_{i}\right)$$

The smoothing regularization term is calculated as

(8) $$L_{smooth}=-\frac{1}{C}\sum_{i=1}^{C}\log\left(p_{i}\right)$$

Finally, the overall training objective is formulated as

(9) $$L_{total}=\left(1-\varepsilon \right)L_{nll}+\varepsilon L_{smooth}$$

where $L_{total}$ is the final optimization objective. By introducing this regularization strategy, DSGF-Net can avoid excessive confidence on training samples and improve robustness under the cross-subject fNIRS classification scenario.

### 3.6. Preprocessing

MI preprocessing method: According to the original study [12], the sampling rate was set to 10 Hz to reduce data redundancy and computational complexity. Subsequently, based on the modified Beer–Lambert Law, the optical density signal was converted into signals representing changes in the concentrations of oxygenated hemoglobin (HbO) and deoxygenated hemoglobin (HbR), which were used to characterize cerebral hemodynamic activity [31]. To reduce noise interference, a 0.01–0.1 Hz bandpass filter was applied to the signal to suppress high-frequency physiological noise (such as heart rate and respiration-related components) and low-frequency drift components [8]. Baseline correction was then performed by subtracting the average signal value within the [−5,−2]-s time interval to mitigate the impact of baseline differences between trials. During the data segmentation phase, a sliding window method was used to segment the continuous signal. The window length was set to 3 s with a step size of 1 s [32], dividing the time interval [−2,10] s to obtain multiple overlapping time segments. The fNIRS classification task does not rely on information from a single time point; rather, the model learns the overall temporal patterns within a window as well as the joint response characteristics across different channels. Therefore, this strategy was used to augment the training dataset under conditions of limited trial data while preserving local temporal dynamics. Training and test subjects were divided according to the LOSO strategy, and sliding-window segmentation was then performed separately within the training and test sets. This ensured that test subject data did not participate in the model training process in any form, thereby avoiding data leakage issues caused by the sliding window. After window segmentation, each subject’s dataset contained 600 samples, generated from 30 trials with 10 temporal windows per trial across two classes. Finally, all samples underwent z-score normalization. The normalization parameters were calculated solely from the training set; that is, the training data were normalized using the mean and standard deviation of the training set, and the test data were processed using the same parameters to reduce the impact of inter-individual amplitude differences and improve the convergence stability of model training.

UFFT Preprocessing Method: For the UFFT dataset, the preprocessing workflow was generally consistent with that of the MI dataset, but there were differences in sampling rate and temporal structure. According to the original study [13], the sampling rate was set to 13.3 Hz. Subsequently, based on the modified Beer–Lambert law, the optical density signals were converted into signals representing changes in HbO and HbR concentrations [31]. Signal preprocessing also employed a 0.01–0.1 Hz bandpass filter to remove high-frequency physiological noise and low-frequency drift components [8]. Since the experimental procedures and stimulus timing settings for the UFFT task differ from those of the MI dataset, this study used the time interval [−1, 0] s as a baseline reference and performed baseline correction by subtracting the average value for this time interval. Baseline correction was then performed by subtracting the average value for the [−1,0]-s time interval to reduce the impact of baseline differences between trials. During the data segmentation phase, a sliding window strategy with a 3-s window and a 1-s step size was used to segment the [0,10]-s time interval, thereby obtaining multiple time-slice samples. Similar to the MI dataset, this operation is performed after trial-level segmentation to avoid data leakage issues. In the UFFT dataset, each subject comprises 600 samples, generated from 25 trials with 8 overlapping windows per trial across three motor-task categories. Finally, all samples are z-score normalized to reduce individual variability and enhance model training stability.

## 4. Experiment

### 4.1. Selection of Datasets

MI dataset: In the MI task, participants were asked to perform kinesthetic motor imagery, specifically imagining themselves grasping a ball (opening and closing their palms). There were 29 participants, each completing a total of 60 trials, including 30 trials of left-hand motor imagery (LHMI) and 30 trials of right-hand motor imagery (RHMI). The procedure for each trial was as follows: (1) An arrow pointing left or right appeared in the center of the screen. (2) The arrow disappeared, accompanied by a beep, a fixation cross appeared, and the participant began the task. (3) The task ended with a beep and the word “STOP”. (4) During rest periods, a fixation cross was displayed, and participants maintained fixation to minimize eye movements. This is a mixed dataset; we only used the fNIRS data from it [12].

UFFT dataset: There were 30 participants, each completing a total of 75 trials, including 25 left-hand tapping (LHT) trials, 25 right-hand tapping (RHT) trials, and 25 foot tapping (FT) trials. During the experiment, participants performed three motor tasks according to on-screen instructions and were instructed to relax during rest periods [13].

**Figure 4:**
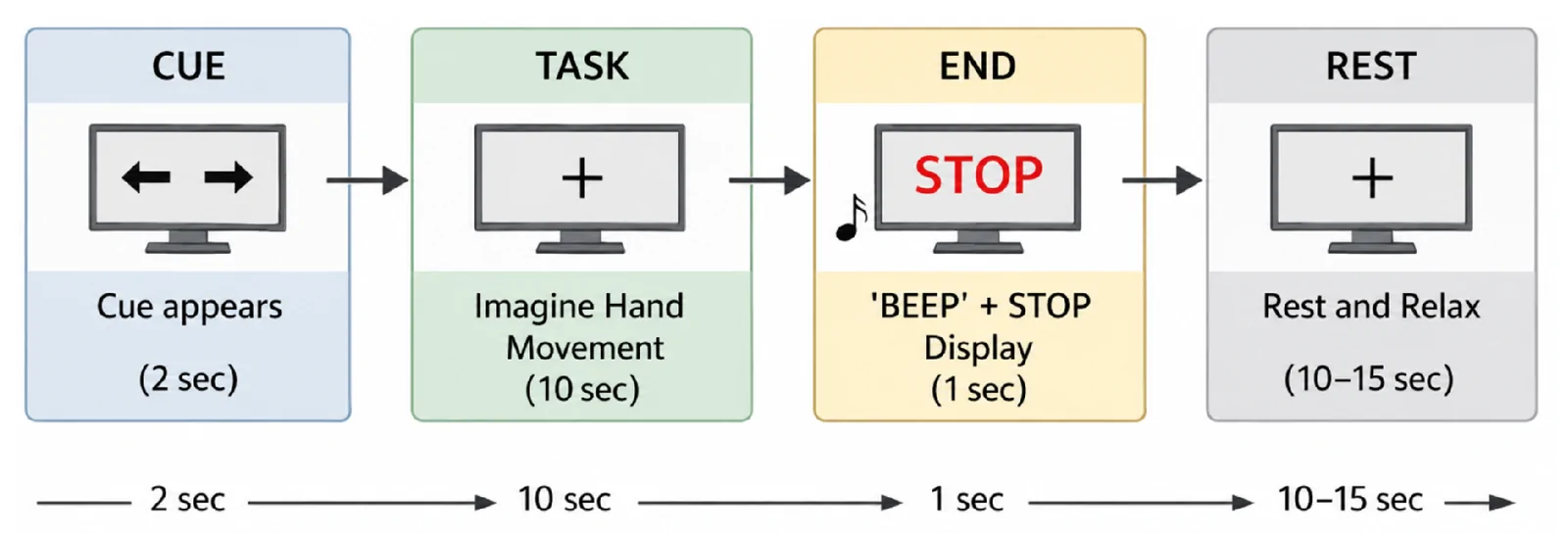
Experimental setup diagrams. The figure shows the experimental setup for the MI dataset.

**Figure 5:**
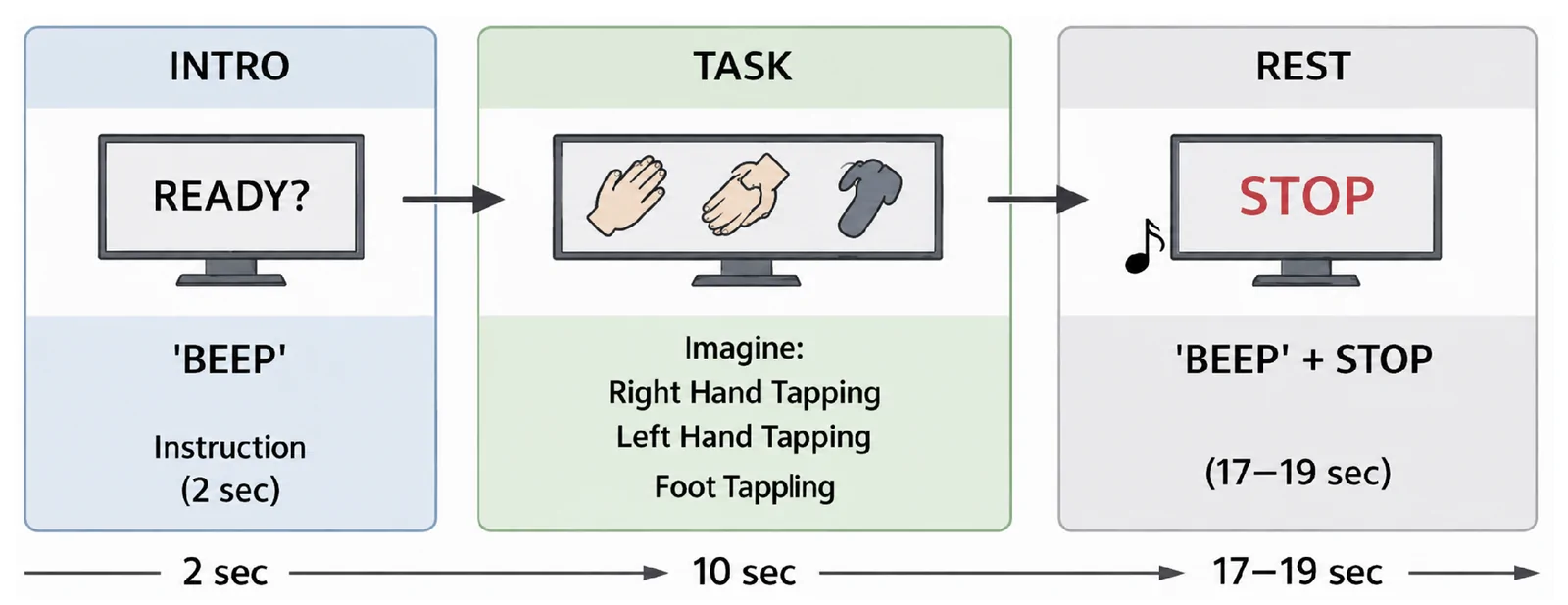
Experimental setup diagrams. The figure shows the experimental setup for the UFFT dataset.

The experimental paradigms for a single trial in the two publicly available datasets we used are shown in Figure 4 and Figure 5.

### 4.2. Evaluation Method

At the experimental design level, this study abandoned the traditional random assignment strategy and instead adopted a “subject-independent” (SI) evaluation paradigm [32], on the basis of which it implemented leave-one-subject-out (LOSO) cross-validation. Specifically, in each iteration, this method completely and independently allocated the data from a single subject to the test set, while the data from all other subjects formed the training set, cycling through all subjects. This rigorous partitioning effectively avoided subject-specific biases and accurately reflected the model’s adaptability and classification performance for unknown subjects. In terms of performance metrics, we used a multi-dimensional set of indicators for a comprehensive evaluation. The key indicators included

Precision:

(10) $$P=\frac{TP}{TP+FP}$$

Recall rate:

(11) $$R=\frac{TP}{TP+FN}$$

F1-score:

(12) $$F1=\frac{2\times P\times R}{P+R}$$

Here, TP stands for true positive, FP for false positive, and FN for false negative.

Kappa coefficient:

(13) $$K=\frac{p_{o}-p_{e}}{1-p_{e}}$$

Here, $p_{o}$ represents the probability of actual agreement among observers, while $p_{e}$ represents the probability of agreement among observers due to chance.

### 4.3. Experimental Setup

This study adopted leave-one-subject-out (LOSO) cross-validation to evaluate the model’s cross-subject generalization ability [33]. In each fold, all trials from one subject were exclusively used as the test set, while the remaining subjects were used for training. To prevent data leakage, data splitting was performed at the subject/trial level before sliding-window segmentation, and the segmentation was conducted separately for the training and test sets. For Z-score normalization, the mean and standard deviation were calculated only from the training data and then applied to the corresponding test subject.

The proposed DSGF-Net was implemented using Python 3.13. The model was trained with AdamW [34] using an initial learning rate of $1\times 10^{-3}$, weight decay of $1\times 10^{-3}$ [35], a batch size of 64, and 50 epochs. Dropout with a rate of 0.5 and label smoothing with a coefficient of 0.1 were employed for regularization. A Cosine Annealing scheduler with $T_{\max}=30$ was used for learning-rate adjustment [36]. For fair comparison, CNN, LSTM, fNIRS-T, and fNIRSNet are re-implemented and re-trained according to their corresponding references rather than directly using previously reported results. All models use the same preprocessing procedure, LOSO data partitioning, optimizer, training epochs, batch size, learning-rate setting, and evaluation protocol. Model-specific architectural parameters are retained according to the requirements of each model. All experiments are conducted using GPU acceleration when available, and model parameters and training logs are saved for reproducibility.

## 5. Analysis of Results

### 5.1. Comparison with Other Models

To validate the effectiveness and generalization ability of the proposed DSGF-Net model, this paper conducts a systematic evaluation using leave-one-subject-out (LOSO) cross-validation on the MI and UFFT datasets and compares it with representative methods such as CNN [15], LSTM [15], fNIRS-T [19], and fNIRSNet [23]. The experimental results for each model on the two datasets are shown in Table 1. On the MI dataset, DSGF-Net achieved the best overall performance across all evaluation metrics (Wilcoxon signed-rank test, *p* < 0.001). Specifically, its classification accuracy reached 62.73%, representing an 8.41 percentage point improvement over the CNN (54.32%) and a 3.17 percentage point improvement over fNIRSNet (59.56%). At the same time, DSGF-Net outperformed the comparison methods in precision (61.95%), recall (62.02%), and F1-score (61.98%), indicating stable performance in class discrimination. The Kappa coefficient reached 0.26, which is also an improvement over other methods, demonstrating that the model exhibits superior consistency in cross-subject evaluations. On the UFFT dataset, DSGF-Net also achieved the best overall results (Wilcoxon signed-rank test, *p* < 0.005). Its accuracy reached 68.48%, an improvement of 11.04 percentage points over the CNN (57.44%) and approximately 4.05 percentage points over fNIRSNet (64.43%). At the same time, it achieved the best performance in precision (69.87%), recall (68.48%), and F1-score (66.72%), with a Kappa coefficient of 0.51, further demonstrating that this method possesses relatively stable predictive performance under more complex task settings. It is worth noting that under the LOSO evaluation setting, significant inter-subject variability exists, making it more difficult for the model to learn feature representations that are consistent across subjects. Under these conditions, the overall performance of traditional CNN and LSTM methods is relatively limited, with accuracy typically below 60%. This phenomenon indicates that relying solely on shallow local features or a single temporal modeling method has certain limitations in the task of classifying fNIRS signals across subjects. In contrast, DSGF-Net extracts complementary features through a dual-branch architecture and combines them with a lightweight gated fusion mechanism to achieve adaptive integration of different feature sources, thereby achieving more stable overall performance on both datasets. Experimental results show that this method demonstrates consistent performance advantages across different task settings, indicating its good adaptability and robustness in cross-subject fNIRS signal classification tasks.

### 5.2. Ablation Studies

To further validate the effectiveness of each component module in DSGF-Net and the role of the gated fusion mechanism in the integration of dual-stream features, we conducted systematic ablation experiments on the MI and UFFT datasets. All ablation experiments in this paper employed the leave-one-subject-out (LOSO) cross-validation strategy, consistent with the main experiments. The experiments primarily included single-branch architectures (LMT Stream and SH Stream), simple feature concatenation (Dual Stream-Concat), and the complete dual-stream fusion model (DSGF-Net). Among these, LMT Stream and SH Stream retain all internal structures within their respective original branches; their output features are fed directly into the final classifier without passing through the GFM, serving to evaluate feature representation capabilities under the bias of a single structure; Dual Stream-Concat retains both branches and concatenates the features from both streams directly before feeding them into the classifier, without using GFM; DSGF-Net, on the other hand, introduces GFM based on the same dual-stream feature extraction architecture. All model architectures were determined prior to the start of the experiment, and the same data splitting method and training strategy were adopted to ensure fairness in comparisons between different models. The experimental results are shown in Table 2.

Experimental results show that, on both datasets, fusing two feature extraction branches yields better classification performance than single-branch models. On the MI dataset, Dual Stream-Concat achieved a performance improvement of approximately 1.47% over SH Stream, the best-performing single-branch model, while DSGF-Net further increased the accuracy to 62.73%. On the UFFT dataset, Dual Stream-Concat achieved an improvement of approximately 1.69% over SH Stream, while DSGF-Net further increased the accuracy to 68.48%. These results indicate that although both LMT Stream and SH Stream incorporate spatiotemporal information modeling, they possess different structural induction biases, enabling them to learn complementary feature representations from distinct perspectives.

A further comparison between Dual Stream-Concat and DSGF-Net reveals that, while keeping the two feature extraction branches completely identical, the introduction of a gated fusion mechanism further improves the model’s performance. On the MI dataset, DSGF-Net achieves an improvement of approximately 1.35% compared to simple feature concatenation; on the UFFT dataset, the improvement is approximately 2.66%. Since the two models differ only in their feature fusion methods, these results directly reflect the additional contribution of GFM to dual-stream feature fusion. These findings suggest that simply combining features from different sources in a fixed manner may not adequately address redundancy or differences in contribution across feature dimensions. In contrast, the gated fusion mechanism dynamically adjusts the importance of different feature dimensions based on the characteristics of the input samples, thereby enhancing the model’s adaptability to signal variations across participants. Since the two models differ only in their feature fusion methods, this result directly reflects the additional contribution of GFM to dual-stream feature fusion. In summary, because the two streams have different structural biases, they can generate complementary feature representations; compared to simple feature concatenation, the GFM module further enables adaptive selection at the feature level, allowing the model to enhance discriminative feature information while suppressing redundant features that may result from individual differences and noise.

### 5.3. Visual Analysis

**Figure 6:**
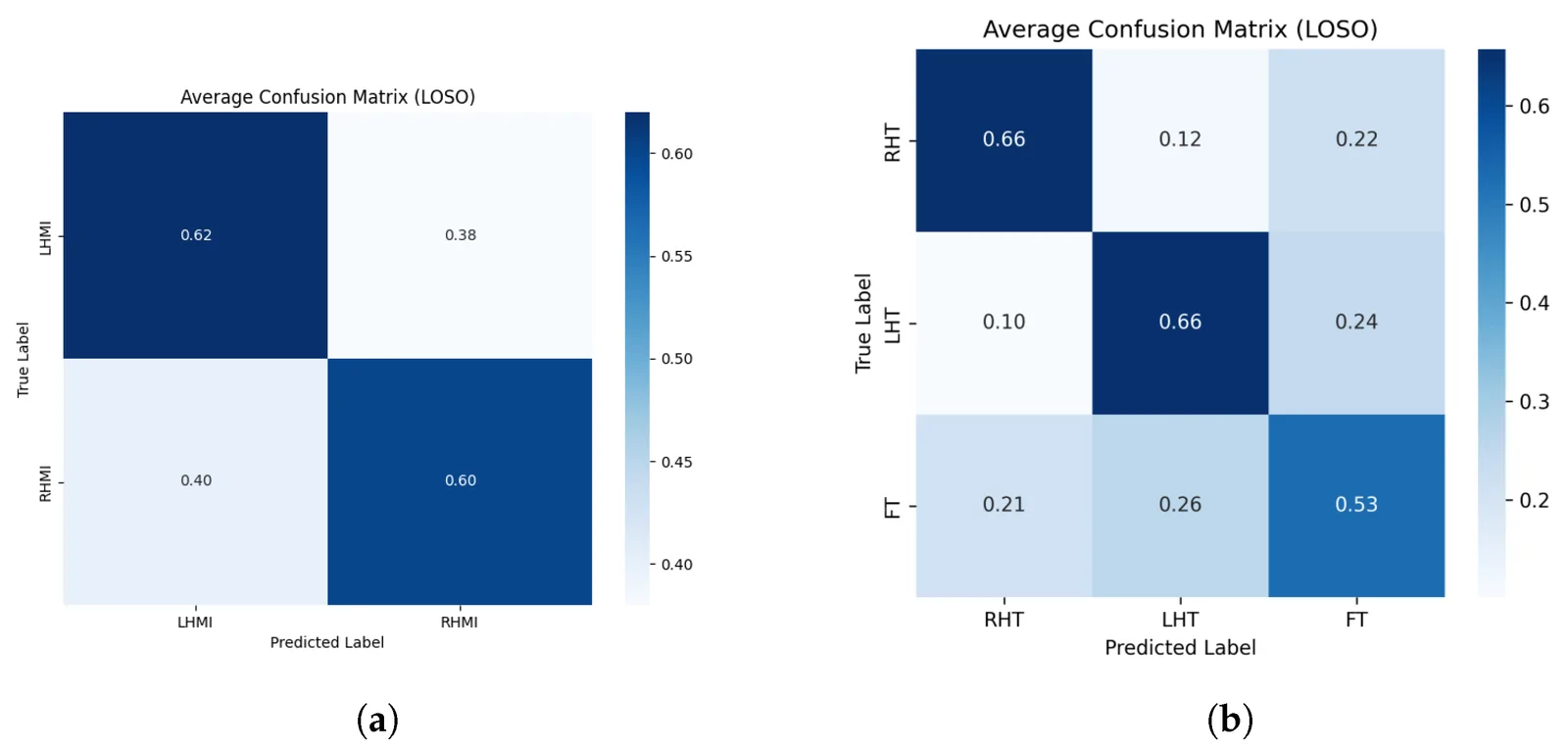
Average confusion matrices of DSGF-Net on the MI and UFFT datasets. (**a**) shows the classification results for left-hand motor imagery (LHMI) and right-hand motor imagery (RHMI) on the MI dataset. (**b**) shows the classification results for right-hand tapping (RHT), left-hand tapping (LHT), and foot movement (FT) on the UFFT dataset. The values represent normalized probability distributions.

**Figure 7:**
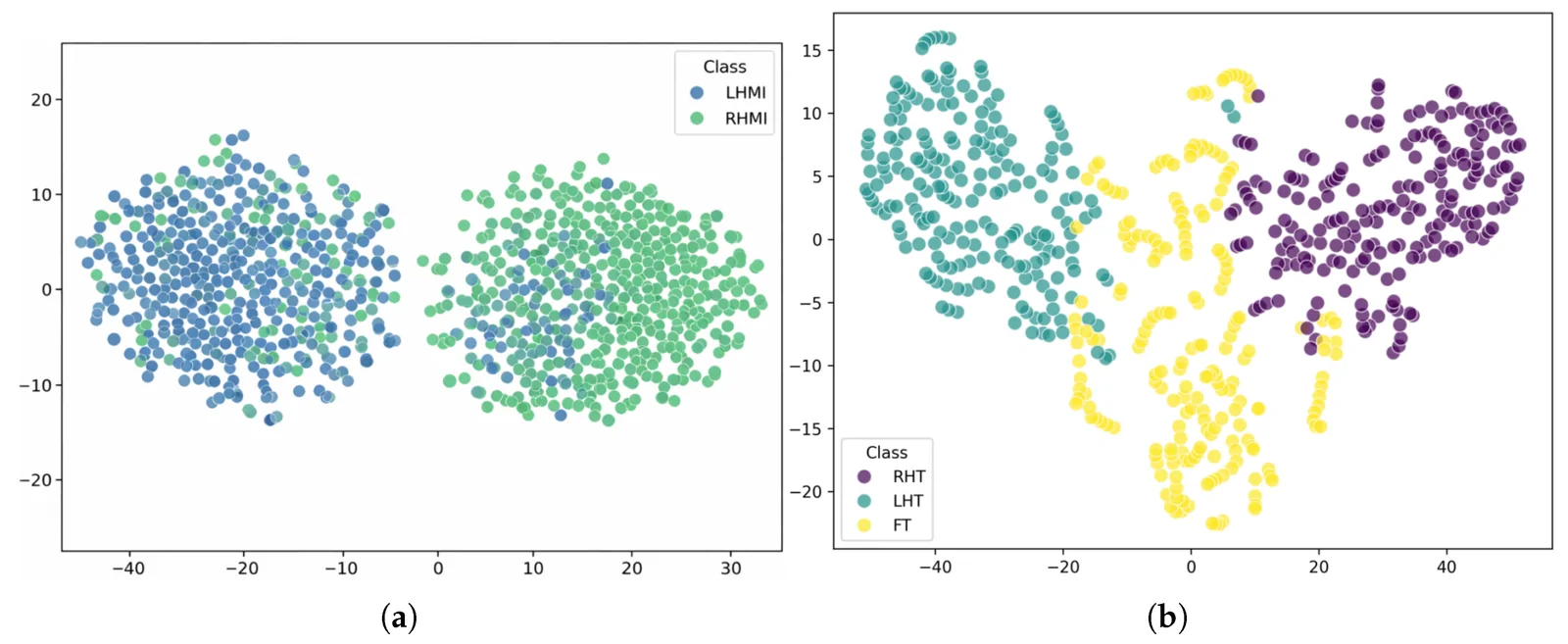
T-SNE visualization of features extracted from representative subjects by DSGF-Net on the MI and UFFT datasets. Each point in the figure represents a sample, and different colors denote different motor task categories. (**a**) MI dataset: Blue represents left-hand motor imagery, and green represents right-hand motor imagery. (**b**) UFFT dataset: Purple represents right-hand tapping, cyan represents left-hand tapping, and yellow represents foot movement.

To further analyze the proposed DSGF-Net from the perspectives of class discrimination performance and feature space distribution, this paper visually presents the average confusion matrix results for all participants and the T-SNE feature distribution for a single representative participant [37], as shown in Figure 6 and Figure 7.

First, the prediction results for all test subjects in the leave-one-out cross-validation (LOSO) were aggregated and averaged to obtain an overall confusion matrix, in order to evaluate the model’s stability in class discrimination across different subjects. As shown in Figure 6, on the MI dataset, DSGF-Net demonstrated relatively stable classification performance for both the LHMI and RHMI tasks, with average classification accuracies of approximately 0.62 and 0.60, respectively. The elements on the main diagonal of the confusion matrix were generally higher than the off-diagonal elements, indicating that the model is able to learn discriminative features to distinguish between left- and right-hand motor imagery tasks to some extent. However, a certain degree of misclassification still existed between the two task categories, which was related to the individual variability of fNIRS signals and the similarity in activation patterns between the left and right hemispheres. As shown in Figure 6, on the UFFT dataset, the model performed relatively well in distinguishing between the RHT and LHT categories, with an average correct classification rate of approximately 0.66. In contrast, the classification accuracy for the FT category was approximately 0.53, and it was more prone to being misclassified as either RHT or LHT. This relatively low classification performance may be related to the spatial characteristics of the cortical regions associated with foot movement, as well as the limited spatial resolution and channel coverage of fNIRS measurements. Compared with hand-related motor areas, foot-related motor areas are primarily located in the more medial regions of the primary motor cortex, where the corresponding hemodynamic responses may be more susceptible to the limited coverage and spatial resolution of the fNIRS probe configuration. Consequently, foot-related neural activity may not be represented as sufficiently and consistently as hand-related activity under the current fNIRS acquisition setup. In addition, the hemodynamic responses associated with FT may exhibit greater inter-subject variability and may partially overlap with the spatial or hemodynamic response patterns of hand-related tasks, thereby increasing the difficulty of cross-subject classification under the LOSO setting. Therefore, the relatively high misclassification rate of the FT category may not be solely attributable to the model itself, but may also reflect the limited spatial representation of foot-related cortical activity by fNIRS and the similarity of hemodynamic response patterns across different motor tasks. Overall, the average confusion matrix results across all participants show that DSGF-Net maintains relatively stable classification performance across multiple categories under the LOSO setting; however, the separability between different categories remains limited by the inherent complexity of the task signals.

To further analyze the distribution of the features learned by the model in the embedding space, we used T-SNE to perform a qualitative analysis. In this paper, we selected one representative subject from each of the MI and UFFT datasets, extracted the features resulting from the final model gating and fusion, and used T-SNE to map them onto a two-dimensional space for visualization. As shown in Figure 7, in the MI dataset, the LHMI and RHMI samples exhibit a certain degree of clustering in the feature space. Overall, the distribution trends between different categories are relatively clear, but local overlap still exists in the boundary regions. This indicates that the model can learn discriminative features with a certain degree of distinguishability, but some confusion still exists for samples located at cross-category boundaries. As shown in Figure 7, in the UFFT dataset, samples from different categories generally exhibit a relatively separated distribution structure in the embedding space. The RHT and LHT categories display a relatively compact clustering structure, while the FT category is distributed more dispersedly and overlaps to some extent with other categories in space. This result indicates that the separability of different task categories in the feature space varies, with the FT category being relatively more difficult to distinguish. It should be noted that these T-SNE results are based on data from a single representative subject and are primarily used for qualitative analysis of the model’s feature representation capabilities, rather than as a direct reflection of overall classification performance. This is consistent with the relatively high classification error rate for the FT class in the confusion matrix, providing an intuitive illustration of the difficulty of classifying this category from the perspective of feature distribution. Therefore, these results should be interpreted in conjunction with the cross-subject statistical results from the confusion matrix.

### 5.4. Comparison of Parameter Values

**Figure 8:**
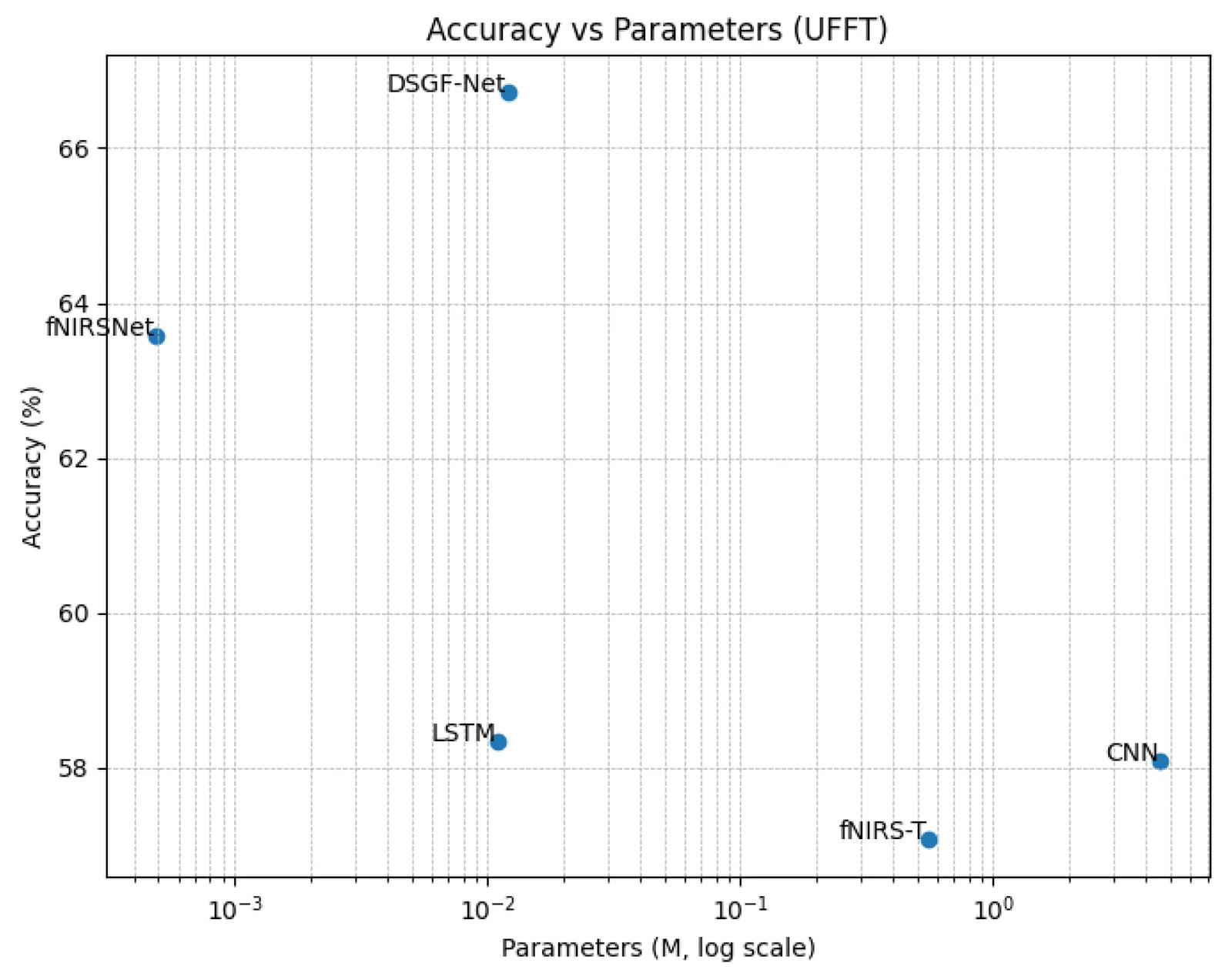
Performance–complexity trade-off on the UFFT dataset.

All models were tested using actual PyTorch networks and analyzed for computational complexity using the THOP tool. For each model, we first constructed a random input tensor with the same dimensions as the actual input and set the batch size to 1. Table 3 presents a comparison of the number of parameters and computational complexity (FLOPs) for different models on the MI dataset [38]. The comparison focuses on the baseline models evaluated in this study, including conventional deep learning models and fNIRS-specific architectures, with the aim of examining the performance–complexity characteristics of DSGF-Net under the same experimental setting. To further illustrate the relationship between model performance and complexity, this paper also provides a plot of accuracy versus the number of parameters on the UFFT dataset (as shown in Figure 8). As shown in the statistical results in Table 3, the CNN model has high computational complexity, with 4.54M parameters and 14.96M FLOPs, while the LSTM and fNIRSNet models have relatively low parameter counts and computational overhead. Among them, fNIRSNet has the lowest number of parameters at 0.49K, but its classification performance is relatively limited. In contrast, DSGF-Net has 12K parameters and 1M FLOPs, which remains substantially smaller than the CNN and fNIRS-T while providing higher classification accuracy in the experiments presented in this study. As further illustrated in Figure 8, on a logarithmic scale of parameter size, DSGF-Net is located in the “lower complexity–higher accuracy” region; within the set of models considered in this study, it achieves a favorable performance–complexity relationship. The design objective of the DSGF-Net model is not merely to minimize the number of parameters, but to reduce computational requirements while maintaining competitive classification performance for cross-subject fNIRS classification. From this perspective, DSGF-Net provides a favorable trade-off among the evaluated fNIRS-related baseline models. Although fNIRSNet is lighter in terms of parameter scale, its classification performance is relatively low; while the CNN and fNIRS-T, although they have certain performance advantages, incur significantly higher computational costs. Through its dual-stream feature extraction architecture and lightweight gated fusion mechanism, DSGF-Net enhances classification performance without introducing the substantially larger parameter and computational costs observed in conventional CNN-based architectures. Therefore, the overall comparison results demonstrate that DSGF-Net achieves a favorable balance between recognition performance and model complexity within the scope of the evaluated models, offering greater potential for practical applications.

## 6. Conclusions

This paper proposes a Dual-Stream Gated Fusion Network, DSGF-Net, for a functional near-infrared spectroscopy (fNIRS) motor imagery brain–computer interface classification task. To address issues such as strong coupling of multimodal hemoglobin information, complex spatiotemporal features, and significant inter-subject variability in fNIRS signals, this paper designs a deep learning framework consisting of two complementary feature extraction branches and a dynamic fusion mechanism to enhance classification performance and model robustness across subjects. Specifically, the model employs a dual-branch structure to perform multi-angle feature modeling of fNIRS signals, with one branch focusing on the extraction of multi-scale temporal dynamic features and the other branch used to learn the spatial distribution patterns of hemodynamic responses across channels. After these two types of features are concatenated in high-dimensional space, they undergo adaptive weighted fusion via a gating mechanism, thereby dynamically adjusting the contribution of different features to enhance discriminative information and suppress interference from redundant features. It should be noted that the dual-branch structure in this study is not a strict spatiotemporal decoupling structure, but rather a complementary feature extraction strategy based on different receptive fields, with the core objective of enhancing the diversity and robustness of feature representations. Experimental results on two public datasets, MI and UFFT, demonstrate that under a leave-one-subject-out (LOSO) cross-validation setting, the proposed method achieves consistent improvements in metrics such as classification accuracy, F1-score, and Kappa coefficient compared to baseline methods including the CNN, LSTM, and fNIRSNet. At the same time, ablation experiments further validated the contributions of the dual-branch structure and the gated fusion mechanism to the overall performance improvement, indicating good complementarity among the various modules. Furthermore, an analysis of model complexity shows that DSGF-Net maintains a low level of parameters and computational overhead; it improves classification performance without significantly increasing the computational burden, demonstrating an optimal performance–complexity balance. Statistical significance analysis further indicates that the proposed method achieves stable and consistent performance improvements compared to the main comparison models, validating its reliability and generalization ability.

Despite achieving certain performance improvements, this study still has some limitations. For example, the current model primarily relies on fixed sliding windows for signal segmentation and has not yet fully accounted for the temporal variability of hemodynamic responses across individuals; furthermore, the model’s stability under conditions of sparse data or cross-dataset transfer remains to be further investigated. Future work will focus on optimizing model performance through adaptive temporal modeling, cross-domain transfer learning, and more refined integration of physiological priors. DSGF-Net provides an effective, lightweight deep learning solution for fNIRS-based motor imagery brain–computer interfaces (BCIs). By enhancing cross-subject classification performance while maintaining control over model complexity, it offers a promising technical pathway for practical BCI system applications.

Future research could explore the following directions: First, conduct validation using larger-scale, multicenter datasets and a wider range of task paradigms to further evaluate the model’s generalization ability; second, address inter-subject variability by introducing domain adaptation, personalized calibration, or self-supervised representation learning strategies, thereby further enhancing the model’s robustness toward unknown subjects; third, constructing a multimodal fusion framework by integrating other neural signals (such as EEG) to fully leverage the complementary information across different modalities and further expand the model’s potential in EEG-fNIRS hybrid brain–computer interface (BCI) applications; fourth, further optimizing the lightweight network architecture and inference efficiency. Overall, DSGF-Net provides an effective and lightweight deep learning solution for fNIRS-based motor imagery brain–computer interfaces (BCIs). By improving cross-subject classification performance while controlling model complexity, this method offers a promising technical path for practical BCI system applications.

## Figures and Tables

**Table 1:** Comparison of evaluation metrics with other models.

Dataset	Model	Accuracy (%)	Precision (%)	Recall Rate (%)	F1 (%)	Kappa
MI	CNN [15]	54.32 ± 7.05	46.03 ± 14.28	57.24 ± 24.72	51.04	0.11
	LSTM [15]	54.29 ± 6.53	54.53 ± 6.73	53.01 ± 7.08	53.76	0.11
	fNIRS-T [19]	55.26 ± 6.17	55.06 ± 6.34	58.56 ± 9.08	56.75	0.14
	fNIRSNet [23]	59.56 ± 5.72	60.37 ± 7.43	60.82 ± 9.06	60.59	0.21
	DSGF-Net	62.73 ± 4.84	61.95 ± 8.27	62.02 ± 10.01	61.98	0.26
UFFT	CNN [15]	57.44 ± 13.76	58.76 ± 13.96	57.44 ± 13.76	58.09	0.35
	LSTM [15]	57.49 ± 13.83	59.23 ± 13.01	57.49 ± 13.83	58.35	0.35
	fNIRS-T [19]	56.86 ± 13.15	57.30 ± 12.93	56.86 ± 13.03	57.08	0.36
	fNIRSNet [23]	63.83 ± 15.29	65.43 ± 15.62	63.83 ± 15.29	64.62	0.46
	DSGF-Net	68.48 ± 15.02	69.87 ± 15.21	68.48 ± 15.02	66.72	0.51

**Table 2:** Performance ablation results for DSGF-Net and its submodules.

Dataset	Model	Accuracy (%)
MI	LMT Stream	57.46 ± 6.05
	SH Stream	59.91 ± 6.65
	Dual Stream-Concat	61.38 ± 5.86
	DSGF-Net	62.73 ± 4.84
UFFT	LMT Stream	58.49 ± 13.42
	SH Stream	64.13 ± 15.27
	Dual Stream-Concat	65.82 ± 14.96
	DSGF-Net	68.48 ± 14.82

**Table 3:** Comparison of parameter counts across different models complexity.

Model	Parameters	FLOPs
CNN [15]	4.54M	14.96M
LSTM [15]	10.91K	0.82M
fNIRS-T [19]	0.54M	13.5M
fNIRSNet	0.49K	10.46K
DSGF-Net	12K	1M

## Data Availability

MI dataset at https://doi.org/10.1109/TNSRE.2016.2628057; UFFT dataset at https://doi.org/10.3390/electronics8121486.
